# Considerations and Challenges in the Application of Large Language Models for Patient Complaint Resolution

**DOI:** 10.2196/65527

**Published:** 2024-09-17

**Authors:** Bin Wei, Xin Hu, XiaoRong Wu

**Affiliations:** 1 The 1st Affiliated Hospital Jiangxi Medical College Nanchang University Nanchang China

**Keywords:** ChatGPT, large language model, LLM, artificial intelligence, AI, patient complaint, empathy, efficiency, patient satisfaction, resource allocation

We are writing to express our appreciation for the recent publication of the study titled “Performance of Large Language Models in Patient Complaint Resolution: Web-Based Cross-Sectional Survey” in the *Journal of Medical Internet Research* [1]*.* The authors provide an innovative perspective on the application of large language models (LLMs) like ChatGPT in resolving patient complaints, demonstrating their potential to enhance patient satisfaction through thoughtful and well-constructed responses. Although we greatly appreciate this study’s meticulous work and significant contributions, we would like to highlight additional considerations and potential limitations for future research.

First, the decision-making process of LLMs is often a “black box.” While these models can generate reasonable and satisfactory responses, the underlying logic and reasoning remain unknown [2]. This lack of transparency and interpretability could pose challenges to the legal responsibilities of health care institutions and potentially undermine patient trust. Additionally, when artificial intelligence (AI) handles sensitive patient information, even with deidentification measures, ensuring data security and privacy remains a critical concern. Therefore, as the application of LLMs expands, establishing appropriate regulatory frameworks and accountability mechanisms may be necessary.

Second, this study may have been conducted within the English-speaking health care system of Singapore, but patient complaints and requirements in different regions heavily depend on local cultural, legal, and social contexts. LLMs may struggle to capture and differentiate these subtle cultural nuances, potentially leading to responses that are not fully appropriate or that even cause misunderstandings. Moreover, while AI models can generate responses that appear empathetic, such responses are merely based on the model’s imitation of language patterns, rather than a true understanding of the patient’s psychological state and emotional needs [3,4]. In the tense environment of doctor-patient relationships in China, if patients perceive a difference in responses, it could exacerbate their anxiety and dissatisfaction. In certain circumstances, using AI to handle patient complaints may lack the necessary human touch, thereby intensifying conflict between patients and health care providers.

Third, over time, LLMs need continuous updates to reflect the latest medical practices, policy changes, and societal expectations, which undoubtedly increases maintenance costs. If AI models generate responses based on incorrect or outdated medical information, they may unintentionally mislead patients, particularly in critical medical decision-making and health guidance, which could be especially dangerous. Furthermore, given the varying levels of health care across different regions, and the differing approaches to treatment, this variability presents a significant challenge for AI.

In conclusion, while LLMs like ChatGPT show great potential in enhancing patient communication and handling complaints, their practical application requires careful consideration of their suitability and the challenges they may present. Therefore, establishing robust regulatory frameworks, ethical guidelines, and stringent oversight mechanisms is crucial. This approach not only helps build trust between patients and health care providers but also contributes to creating a more patient-centered and efficient health care system.

## Abbreviations

AI: artificial intelligence
LLM: large language model
